# Below or all the way to the peak? Oxygen uptake efficiency slope as the index of cardiorespiratory response to exercise—the NOODLE study

**DOI:** 10.3389/fphys.2024.1348307

**Published:** 2024-01-26

**Authors:** Przemysław Kasiak, Tomasz Kowalski, Kinga Rębiś, Andrzej Klusiewicz, Michał Starczewski, Maria Ładyga, Szczepan Wiecha, Marcin Barylski, Adam Rafał Poliwczak, Piotr Wierzbiński, Artur Mamcarz, Daniel Śliż

**Affiliations:** ^1^ 3rd Department of Internal Medicine and Cardiology, Medical University of Warsaw, Warsaw, Poland; ^2^ Doctoral School, Medical University of Warsaw, Warsaw, Poland; ^3^ Department of Physiology, Institute of Sport—National Research Institute, Warsaw, Poland; ^4^ Faculty of Rehabilitation, Jozef Pilsudski University of Physical Education in Warsaw, Warsaw, Poland; ^5^ Faculty of Physical Education and Health, Jozef Pilsudski University of Physical Education in Warsaw, Branch in Biala Podlaska, Biała Podlaska, Poland; ^6^ Department of Internal Medicine and Cardiac Rehabilitation, Medical University of Lodz, Łódź, Poland

**Keywords:** cardiopulmonary exercise test, endurance athletes, exercise physiology, oxygen uptake efficiency slope, prediction equation

## Abstract

**Background:** The ratio of oxygen uptake (VO_2_) to minute ventilation (VE) is described as the oxygen uptake efficiency slope (OUES). OUES has been suggested as a valuable submaximal cardiorespiratory index; however, its characteristics in endurance athletes remain unknown. In this study, we a) investigated OUES between different time intervals, b) assessed their prediction power for VO_2_peak, and c) derived new prediction equations for OUES tailored for well-trained individuals.

**Materials and Methods:** A total of 77 male (age = 21.4 ± 4.8 yrs; BMI = 22.1 ± 1.6 kg·m^−2^; peak oxygen uptake = 4.40 ± 0.64 L·min^−1^) and 63 female individuals (age = 23.4 ± 4.3 yrs; BMI = 23.1 ± 1.6 kg·m^−2^; peak oxygen uptake = 3.21 ± 0.48 L·min^−1^) underwent the cycling cardiopulmonary exercise test. OUES was measured at 75%, 90%, and 100% of exercise duration. Prediction power and new models were derived with the multiple linear regression method.

**Results:** In male subjects, OUES [mL·min^−1^/L·min^−1^] from 75% = 4.53 ± 0.90, from 90% = 4.52 ± 0.91, and from 100% = 4.41 ± 0.87. In female subjects, OUES [mL·min^−1^/L·min^−1^] from 75% = 3.50 ± 0.65, from 90% = 3.49 ± 0.62, and from 100% = 3.41 ± 0.58. OUES did not differ between time intervals in male (*p* = 0.65) and female individuals (*p* = 0.69). OUES strongly predicts peak VO_2_ independently from the measuring interval (*β* = 0.71–0.80; *R* ^2^ = 0.50–0.63). The prediction model designed for elite athletes was OUES [mL·min^−1^/L·min^−1^] = −1.54 + 2.99; BSA [m^2^]—0.0014; (age [in years]; sex [1 = male, 2 = female]) (*R* ^2^ = 0.36).

**Conclusion:** OUES enables an accurate prediction of peak cardiorespiratory fitness in elite endurance athletes. OUES is a feasible alternative to maximal exercise testing. A new prediction equation should be used for highly trained individuals. Physicians should understand OUES physiology to properly assess the cardiorespiratory response to exercise in athletic cohorts.

## Introduction

Maximal symptom-limited cardiopulmonary exercise testing (CPET) is a gold standard of assessment of an individual’s endurance capacity, and its diagnostic value is most often assigned to maximal effort (Balady et al., 2010). However, Baba et al. introduced a new submaximal exercise performance indicator, the oxygen uptake efficiency slope (Baba et al., 1996). OUES has previously been evaluated in a clinical context to stratify the risk for cardiovascular diseases (Hollenberg and Tager, 2000).

OUES is plotted as the course of oxygen uptake (VO_2_) relative to the logarithm of minute ventilation (VE) (Baba et al., 1996). VE is mostly affected by the partial pressure of carbon dioxide and further acidemia, and VO_2_ reflects the oxygen absorption of the body (Baba et al., 1996). Similarly to peak VO_2_ (VO_2_peak), OUES is determined by the integrated functions of several different physiological systems: musculoskeletal, cardiac, and respiratory (Baba et al., 1996). A key advantage of OUES is the stable, linear course throughout the whole exercise duration (Sun et al., 2012). This is possible by the logarithmic transformation of the curvilinear relation of VE to VO_2_ (Akkerman et al., 2010). OUES represents an almost excellent linear course during the whole physical effort, and OUES determines how effectively oxygen is transported (Baba et al., 1996). Previous research has suggested that, in the general population, OUES may correlate well with VO_2_peak and allows for its accurate prediction only with the submaximal CPET (Ashikaga et al., 2021).

OUES enables a comprehensive assessment of the cardiac response to physical exercise (Baba, 2000; Akkerman et al., 2010). Individuals with higher fitness levels had a steeper OUES (Sun et al., 2012). VO_2_ and OUES measure a close physiological mechanism (Hollenberg and Tager, 2000). OUES and VO_2_peak correlate well with each other (Baba et al., 1999a). VO_2_ and OUES increase simultaneously with growing exercise intensity (Baba et al., 1996). Moreover, the linear relationship between OUES and VO_2_ should be maintained independently from the performance achieved during the exercise test (Baba et al., 1996; Baba et al., 1999a). A reduced slope is found in individuals with limited functional capacity or suggested pathology (Baba, 2000; Sun et al., 2012). OUES has been most often determined with 75% (OUES_75_), 90% (OUES_90_), and 100% (OUES_100_) of data from the CPET protocol (Baba et al., 1996; Hollenberg and Tager, 2000). Among normal, healthy populations, all measuring intervals provide comparable values (Hollenberg and Tager, 2000), and OUES also maintains this relationship under clinical conditions (Baba et al., 1999b).

Moderate endurance training has several health benefits (Kim and Baggish, 2017). However, elite athletes are subjected to strenuous physical demands (Wasfy and Baggish, 2016). This results in a higher risk of cardiovascular diseases (CVDs) (Andersen et al., 2013; Schnohr et al., 2015). Athletes usually do not fit well into the general cardiopulmonary reference values (Petek et al., 2021; Wiecha et al., 2023b). Moreover, no studies so far compared the prediction powers of different OUES intervals on VO_2_peak among well-trained endurance athletes (Akkerman et al., 2010). We stipulate that all those relationships could be more complex than in untrained individuals. Therefore, determining the underlying response profile of OUES for the athletic population remains crucial to avoid controversies and misdiagnosis.

In this study, we a) explained the relationship between OUES intervals for endurance athletes, b) assessed the prediction power of OUES for VO_2_peak in well-trained participants, c) derived and internally validated new prediction equations for OUES, and d) externally validated current prediction equations in a highly trained reference cohort.

## Materials and methods

### Study design

We applied the recommendations for observational studies by the EQUATOR Network–STROBE Guidelines for cross-sectional studies (Supplementary Table S1). CPET was performed by endurance athletes in the years 2022–2023. Exercise tests took place at the Institute of Sport–National Research Institute in Warsaw. The study was reviewed and approved by the Bioethics Committee of the Medical University of Warsaw (AKBE/277/2023). Written informed consent was obtained for each subject. In particular, we did not initially refer to the CPET potential study candidates who had past medical or family histories and evident medical conditions (diagnosed during pre-participation physical examination).

**FIGURE 1:**
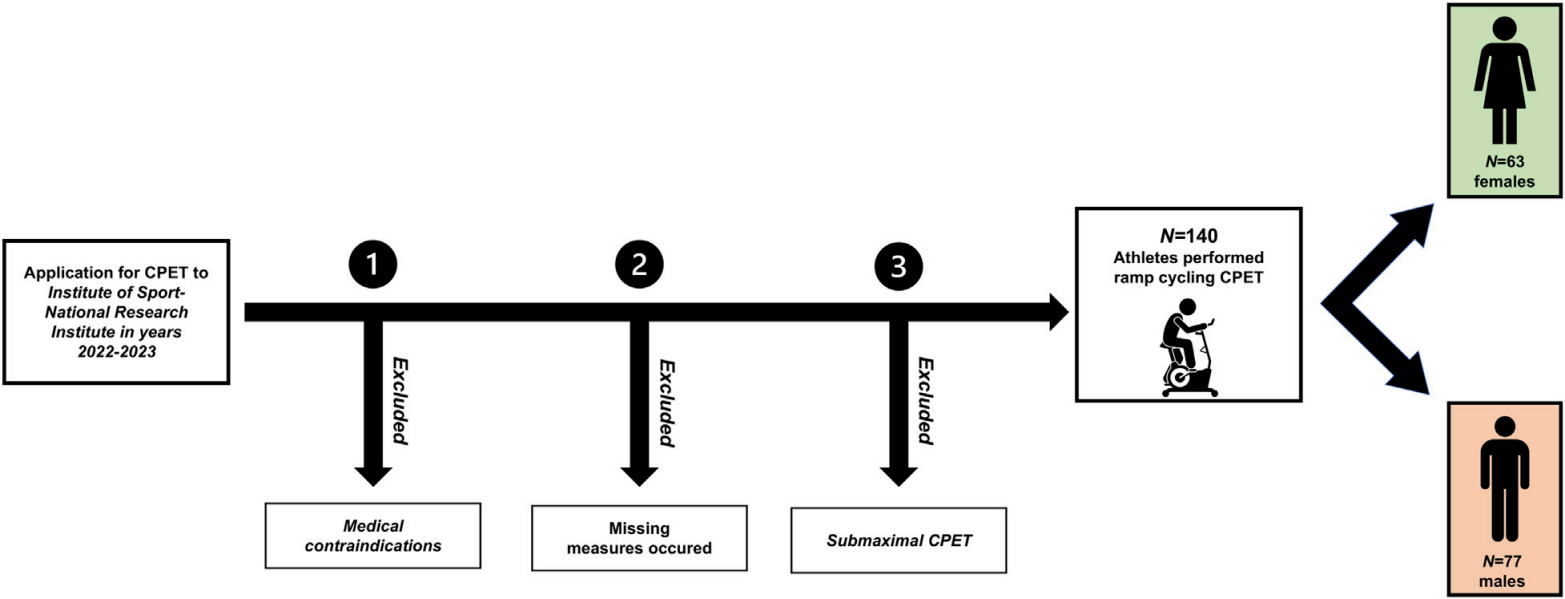
Flowchart for the study recruitment process. Abbreviation: CPET, cardiopulmonary exercise test. Note: only adult endurance athletes were enrolled in the study. A 3-step recruitment process was performed. During the first step, medical contraindications considered as mandatory exclusion criteria were: cardiovascular conditions, respiratory conditions, actual psychiatric or neurological disease, orthopedic conditions restricting effort at CPET, divergences in complete blood count, and tobacco smoking. At the second step, if any missing variables occurred, participants were excluded from the analysis to ensure the highest reliability of the analyses. Finally, in the third step, athletes with submaximal performance were not included, and the peak effort was defined as plateau in oxygen uptake, respiratory exchange ratio ≥1.05, declaration of volitional exhaustion with a rating of perceived exertion ≥18 according to the Borg scale, and maximal heart rate was ≥80% of the age-predicted value.

Only adult subjects of age ≥18 years were included. To be eligible for this study, endurance athletes had to be free from a) cardiovascular or pulmonary conditions, b) psychiatric or neurological diseases, d) orthopedic conditions restricting effort at CPET, e) divergences in complete blood count, f) and tobacco smoking. All athletes had at least a 4-year experience in regular endurance training and were members of a training club and elite or development national teams in Olympic sports. They periodically participated in both national and international competitions, including the Olympic Games. The participants of this study belonged to tiers 3–5, according to the McKay classification system (McKay et al., 2022). The full participant selection process is detailed in Figure 1.

### Cardiopulmonary exercise testing

All endurance athletes performed maximal-graded symptom-limited CPET. We considered the maximal exercise response when there was a a) ≥30-s VO_2_ plateau, b) respiratory exchange ratio (RER) ≥1.05, c) declaration of fatigue confirmed by a Borg’s RPE ≥18, and d) measured heart rate ≥80% of the age-predicted value. Experienced physiologists supervised the CPET, and the athletes were verbally motivated to reach maximal effort.

The athletes underwent ramp cycling CPET on a Cyclus2 Ergometer (RBM, Leipzig, Germany). At the beginning of CPET, there was a brief 2–3 min warm-up of freewheeling pedaling. The initial load began with 55–70 W and was progressively raised by 0.17–0.28 W s^−1^. The resistance was modified individually in provided ranges to adjust the intensity in agreement with each participant.

### Body measurements

We obtained the following anthropometric measurements: age, weight, height, and body mass index (BMI). The weight was measured prior to breakfast by a TANITA weight scale (TANITA Corporation, Arlington Heights, IL, United States). The height was measured in the morning (in the same time as weight) with a stadiometer (Seca GmbH and Co., Hamburg, Germany).

### Cardiopulmonary fitness outcomes

Polar H10 (Polar Electro Oy, Kempele, Finland) was used to monitor the heart rate (HR). Gas-exchange variables were taken breath-by-breath with a V2 Mask (Hans Rudolph, Inc., Shawnee, KS, United States) and a Cortex B3 Metamax system (CORTEX Biophysik GmbH, Leipzig, Germany). All the pieces of equipment used were calibrated according to the manufacturer’s guidelines before each CPET. We applied 15-s intervals to average the measurements. The measured VO_2_peak was compared with the VO_2_peak predicted by the Wasserman and Hansen equation (Wasserman et al., 1987).

OUES was defined according to the method of Baba et al. (1996): VO_2_ = a · log_10_ · VE + b., where “a” represents the OUES. OUES was calculated at 75%, 90%, and 100% of the exercise duration. The first minute of the loaded protocol was not included in the analysis, as recommended by Hollenberg and Tager (2000). The body surface area was calculated using the Du Bois and Du Bois formula: 0.007184 · height (cm)^0.725^ · weight (kg)^0.425^ (Burton, 2008). We additionally calculated the HR (both absolute and percentage of peak HR) for each intensity zone (OUES_75_, OUES_90_, and OUES_100_).

### Prediction models for external validation

Prediction equations for external validation were chosen from the review by Akkerman et al. (2010) up to year 2010 and through additional search for studies published in years 2010–2024. The applied keywords were “oxygen uptake efficiency slope,” “OUES,” “prediction model,” “prediction equation,” “cardiopulmonary exercise testing,” “reference values,” and “linear regression” in the scientific literature. Equations derived from clinical cohorts (with comorbidities) were excluded to ensure maximal similarity. To sum up, 11 reference equations from seven studies fulfilled the inclusion criteria. Their characteristics are provided in Table 1.

**TABLE 1:** Prediction models selected for validation.

Study	Model’s type	Prediction equation		Exercise protocol	Study population [all/female subject/male subject]
		Male subject	Female subject		
Hollenberg and Tager (2000) [mL·min^−1^/L·min^−1^]		1,320–26.7 · age + 1,394 · BSA	1,175–15.8 · age + 851 · BSA	Running CPET; Cornell variation in the Bruce protocol	998/579/419
Buys et al. (2015)	1 [mL·min^−1^/L·min^−1^]	3,930–12.5 · age	3,013–15 · age	Cycling CPET; stepwise protocol with initial workload 20 W and increases of 20 W · min^−1^ until termination	1,411/877/534
	2 [mL·min^−1^/L·min^−1^]	1,093—18.5 · age + 1,479 · BSA	842–18.5 · age + 1,280 · BSA•		
	3 [mL·min^−1^/L·min^−1^]	1897–18.3 · age—631 · sex + 1,394 · BSA•			
Milani et al. (2023)	1 [mL·min^−1^/L·min^−1^]	2,682 + 45.47 · age—0.7658 · age^2^	1,436 + 38.02 · age—0.5565 · age^2^	Running CPET; ramp protocol with an initial speed of 2–4 km · h^−1^ and individually graded increases	3,544/1574/1970
	2 [mL·min^−1^/L·min^−1^ · weight^−1^]	36.80 + 0.2968 · age—0.005726 · age^2^	26.11 + 46.90 · age—0.007472 · age^2^•		
	3 [mL·min^−1^/L·min^−1^ · BSA^−1^]	1,450 + 17.47 · age—0.3011 · age^2^	895.2 + 20.58 · age—0.3043 · age^2^•		
Ashikaga et al. (2021) [mL·min^−1^/L·min^−1^]		2,841–12 · age	2,841–12 · age–753 + 4 · age	Cycling CPET; ramp protocol with increases in the power of 10 W · min^−1^ or 20 W · min^−1^	529/274/255
Sun et al. (2012) [L·min^−1^/L·min^−1^]		−0.610–0.032 · age + 0.023 · height + 0.008 · weight	−0.610–0.032 · age + 0.023 · height + 0.008 · weight - 0.568	Running and/or cycling CPET; ramp protocol with 3-min resting and 3-min warm-up, followed by maximal effort and terminated with at least 2-min recovery	474/136/281
Marinov et al. (2007) [mL·min^−1^/L·min^−1^]		−398 + 1958.1 · BSA	−398 + 1958.1 · BSA—199.5	Running CPET; ramp modified Balke protocol with an initial grade of 6% and increase of 2% · min^−1^ and a fixed speed 5.4 km · h^−1^	114/56/58
Marinov and Kostianev. (2003) [mL·min^−1^/L·min^−1^]		−3,346.9 + 28.08 · height + 794.2 · BSA		Running CPET; ramp modified Balke protocol with an initial grade of 6% and an increase of 2% · min^−1^ and a fixed speed 5.4 km · h^−1^	60/30/30

Abbreviations: BSA, body surface area; CPET, cardiopulmonary exercise test.

Note: age is expressed in years, BSA is calculated in m^2^, weight is presented in kg, and height is shown in cm. Sex was computed as 1 for male subjects and 2 for female subjects.

### Statistical analysis

First, data distribution was evaluated with the Shapiro–Wilk test and quantile–quantile plots. The continuous measures have been shown as mean (standard deviation [±]). The categorical measures have been shown as numbers (percentage [%]). An athlete was removed from the analysis when there were any missing measurements, with the aim of supporting maximal credibility of the results. The significance borderline was set at a two-sided *p*-value < 0.05.

For applied statistical tests, we verified the cohorts’ sizes. The whole population or particular subgroups (males and females) fulfilled the required numbers to achieve a large effect size, statistical significance (*p* < 0.05), and high power (>0.8).

Differences between all measurements (OUES_75_, OUES_90_, and OUES_100_) were compared by one-way ANOVA. Additionally, Student’s t-test for independent means was conducted to compare differences between paired measurements (OUES_75_ and OUES_90_, OUES_75_ and OUES_100_, and OUES_90_ and OUES_100_). Linear regression was used to examine the prediction power of OUES_75_, OUES_90_, and OUES_100_ on VO_2_peak. Each calculation was carried out independently for male and female individuals.

The model’s derivation was preceded by an assessment of the data assumptions (correlations, collinearity, independence of observations, analysis of residuals, and leverage or influence plots). We used multiple linear regression to derive new prediction equations. The performance of new models was presented with the root-mean-square error (RMSE) and two-way mixed-effects interclass correlation coefficient (ICC_3,1_) (Koo and Li, 2016). The Bland–Altman plots and 10-fold cross-validation were used to evaluate the model’s agreement (Jung and Hu, 2015). Linear models were developed to externally validate equations by regressing the predicted OUES against the observed OUES. In analysis, the coefficient of determination (*R* ^2^) was considered as the adjusted *R* ^2^.

The results have been shown in line with the APA style guidelines. IBM SPSS (version 29.0, IBM, Chicago, IL, United States) was used for analysis, and GraphPad Prism (version 10.1.0, GraphPad Software, San Diego, California, United States) was used to prepare figures.

## Results

### OUES relationships among elite athletes

A total of 140 healthy endurance athletes were qualified. A total of 45.0% (n = 63) of the study population were female individuals, and 55.0% (n = 77) of the population were male individuals. The participants represented sports from endurance-oriented Olympic disciplines. A total of 40.0% (n = 56) were specified in triathlon or cycling, 42.1% (n = 59) in speedskating, and 17.9% (n = 25) chose other disciplines. Percent-predicted VO_2_peak equals 144.5% ± 25.9% according to the Wasserman and Hansen equation. The demographic characteristics and exercise results are stratified by sex in Table 2.

**TABLE 2:** Demographic and exercise characteristics.

Measurement		Whole group [n = 140]	Female subject [n = 63]	Male subject [n = 77]
Demographic characteristic				
Age [years]		22.7 ± 4.6	23.8 ± 4.2	21.8 ± 4.8
Height [cm]		174.8 ± 9.9	166.3 ± 6.2	181.6 ± 6.3
Weight [kg]		69.3 ± 10.1	61.0 ± 5.5	76.1 ± 7.6
BMI [kg·m^−2^]		22.6 ± 1.7	22.1 ± 1.6	23.1 ± 1.7
BSA [m^2^]		1.84 ± 0.12	1.68 ± 0.10	1.97 ± 0.18
Sport discipline	Triathlon/cycling	56 (40.0)	30 (47.6)	26 (33.8)
	Speedskating	59 (42.1)	26 (41.3)	33 (42.9)
	Other	25 (17.9)	7 (11.1)	18 (23.3)
Exercise performance				
HRpeak [beats·min^−1^]		190.9 ± 8.9	191.0 ± 9.1	190.8 ± 8.7
VEpeak [L·min^−1^]		154.5 ± 34.1	127.8 ± 21.1	176.3 ± 26.3
VO_2_peak [L·min^−1^]		3.86 ± 0.82	3.21 ± 0.48	4.40 ± 0.64
VO_2_peak [mL·kg^−1^·min^−1^]		55.2 ± 8.6	52.1 ± 7.0	57.8 ± 9.0
VO_2_peak [% predicted]		144.5 ± 25.9	161.4 ± 21.8	130.6 ± 20.2
VCO_2_peak [L·min^−1^]		4.36 ± 0.96	3.57 ± 0.52	5.00 ± 0.72
RRpeak [breaths·min^−1^]		60.0 ± 7.6	60.2 ± 6.7	59.9 ± 8.3
VT [L]		2.81 ± 0.64	2.30 ± 0.32	3.22 ± 0.53
RER [VO_2_/VCO_2_]		1.14 ± 0.05	1.13 ± 0.05	1.15 ± 0.05
O_2_Ppeak [VO_2_/HR]		20.7 ± 4.4	17.3 ± 3.0	23.5 ± 3.3
Exercise time [minutes]		21.3 ± 2.6	21.1 ± 2.7	21.4 ± 2.6
Workload [watts]		320.4 ± 76.2	266.7 ± 40.8	364.4 ± 70.0
OUES_75_ [mL·min^−1^/L·min^−1^]		4.07 ± 0.95	3.50 ± 0.65	4.53 ± 0.90
HR at OUES_75_ [beats·min^−1^]		173.0 ± 10.5	175.1 ± 10.1	171.3 ± 10.5
HR at OUES_75_ [%HRpeak]		90.6 ± 2.7	91.7 ± 2.1	89.8 ± 2.8
OUES_90_ [mL·min^−1^/L·min^−1^]		4.06 ± 0.95	3.49 ± 0.62	4.52 ± 0.91
HR at OUES_90_ [beats·min^−1^]		183.1 ± 9.3	184.5 ± 9.4	182.0 ± 9.1
HR at OUES_90_ [%HRpeak]		95.9 ± 1.4	96.6 ± 1.1	95.4 ± 1.4
OUES_100_ [mL·min^−1^/L·min^−1^]		3.96 ± 0.90	3.41 ± 0.58	4.41 ± 0.87

Abbreviations: BMI, body mass index; BSA, body surface area; HRpeak, peak heart rate; VEpeak, peak minute ventilation; VO_2_peak, peak oxygen uptake; VCO_2_peak, peak carbon dioxide output; RRpeak, peak respiratory rate; VT, tidal volume; RER, peak respiratory exchange ratio; O_2_Ppeak, peak oxygen pulse; OUES_75_, oxygen uptake efficiency slope from 75% of exercise duration; HR, heart rate; OUES_90_, oxygen uptake efficiency slope from 90% of exercise duration; OUES_100_, oxygen uptake efficiency slope from 100% of exercise duration.

Note: values are presented as mean ± standard deviation or number (%). HR for OUES was considered the peak HR, which occurred directly at the end of OUES_75_ and OUES_90_. HR at OUES_100_ was HRpeak. Predicted VO_2_peak has been calculated according to the Wasserman and Hansen equation.

**FIGURE 2:**
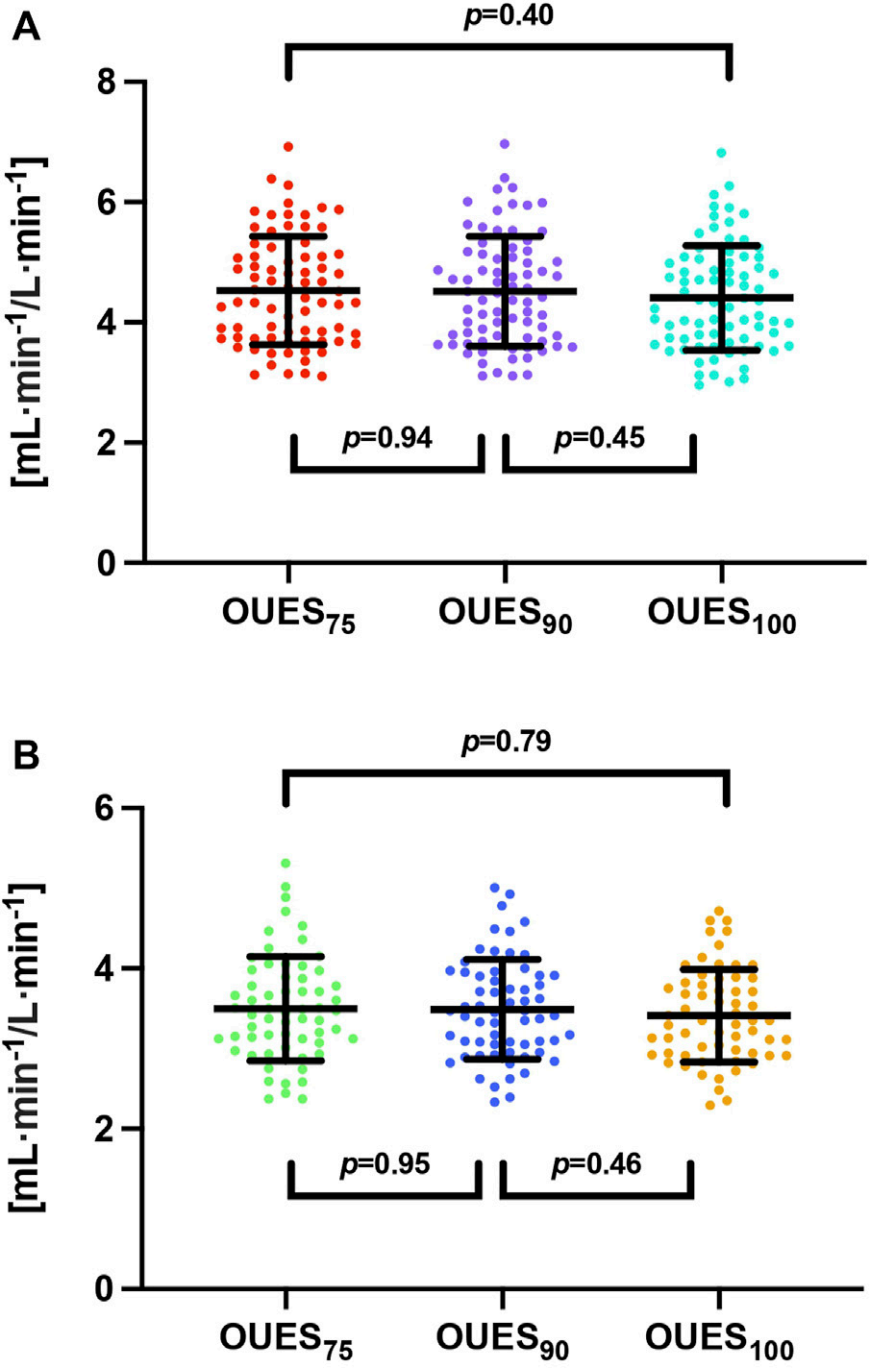
Oxygen uptake efficiency slope variability among endurance athletes. Abbreviations: OUES_75_, oxygen uptake efficiency slope from 75% of exercise duration; OUES_90_, oxygen uptake efficiency slope from 90% of exercise duration; OUES_100_, oxygen uptake efficiency slope from 100% of exercise duration. Note: **(A)** represents male subjects, while **(B)** represents female subjects. There were no significant differences in independent bivariable comparisons or comprehensive multivariable comparisons.

For males, the mean OUES_75_ (4.53 ± 0.90 mL·min^−1^/L·min^−1^) was not significantly different than OUES_90_ [(4.52 ± 0.91 mL·min^−1^/L·min^−1^); t (152) = 0.08, *p* = 0.94] or OUES_100_ [(4.41 ± 0.87 mL·min^−1^/L·min^−1^); t (152) = −0.85, *p* = 0.398]. Similarly, OUES_90_ did not differ significantly from OUES_100_ [t (152) = −0.76, *p* = 0.45]. The same relationship was observed among female subjects. Average OUES_75_ (3.50 ± 0.65 mL·min^−1^/L·min^−1^) did not differ significantly from OUES_90_ [(3.49 ± 0.62 mL·min^−1^/L·min^−1^); t (124) = 0.07, *p* = 0.95] or OUES_100_ [(3.41 ± 0.58 mL·min^−1^/L·min^−1^); t (124) = 0.79, *p* = 0.43]. Moreover, OUES_90_ did not differ significantly from OUES_100_ [t (124) = 0.73, *p* = 0.46]. One-way ANOVA revealed that there was no significant difference in all OUES measurements both for male [F (2,228) = 0.43, *p* = 0.65] and female subjects [F (2,168) = 0.38, *p* = 0.69]. On average, the difference between OUES_75_ and OUES_90_ was 0.11 ± 0.11 mL·min^−1^/L·min^−1^ for male individuals and 0.12 ± 0.12 mL·min^−1^/L·min^−1^ for female individuals, that between OUES_75_ and OUES_90_ was 0.21 ± 0.19 mL·min^−1^/L·min^−1^ for male individuals and 0.18 ± 0.20 mL·min^−1^/L·min^−1^ for female individuals, and that between OUES_90_ and OUES_100_ was 0.13 ± 0.11 mL·min^−1^/L·min^−1^ for male individuals and 0.10 ± 0.12 mL·min^−1^/L·min^−1^ for female individuals. OUES dependencies among the endurance athletes are shown independently for male and female individuals in the lower rows of Table 2 and Figure 2.

### Prediction power of OUES for VO_2_peak

Univariable models were developed to examine OUES prediction power on VO_2_peak. OUES presented a high ability to predict VO_2_peak. OUES explained up to 63% of the variance in VO_2_peak in male subjects and up to 62% of the variance in VO_2_peak in female subjects. VO_2_peak increased simultaneously with OUES calculated from all time intervals. This relationship was more noticeable in male individuals (*β* = 0.73–0.80) than in female individuals (*β* = 0.71–0.79). The RMSE was consistently lower in female individuals than in male individuals. For the presented models, the predicted VO_2_peak is in L·min^−1^ and OUES should be calculated in mL·min^−1^/L·min^−1^.

The fitted univariable models for male subjects were expressed as follows:

(a) VO_2_peak = 2.07 + 0.51 · OUES_75_

The regression model covered 52% of the variance in VO_2_peak (*R* ^2^ = 0.52, F (1, 75) = 83.6, *p* < 0.001), and OUES_75_ significantly predicted VO_2_peak (*β* = 0.73, *p* < 0.001). The RMSE was 0.44 L·min^−1^.

(b) VO_2_peak = 1.97 + 0.54 · OUES_90_

The regression model covered 59% of the variance in VO_2_peak (*R* ^2^ = 0.59, F (1, 75) = 108.2, *p* < 0.001), and OUES_75_ significantly predicted VO_2_peak (*β* = 0.77, *p* < 0.001). The RMSE was 0.44 L·min^−1^.

(c) VO_2_peak = 1.81 + 0.59 · OUES_100_

The regression model covered 63% of the variance in VO_2_peak (*R* ^2^ = 0.63, F (1, 75) = 131.5, *p* < 0.001), and OUES_75_ significantly predicted VO_2_peak (*β* = 0.80, *p* < 0.001). The RMSE was 0.39 L·min^−1^.

The fitted univariable models for female subjects were expressed as follows:

(a) VO_2_peak = 1.36 + 0.53 · OUES_75_

The regression model covered 50% of the variance in VO_2_peak (*R* ^2^ = 0.50, F (1, 61) = 63.0, *p* < 0.001), and OUES_75_ significantly predicted VO_2_peak (*β* = 0.71, *p* < 0.001). The RMSE was 0.34 L·min^−1^.

(b) VO_2_peak = 1.18 + 0.58 · OUES_90_

The regression model covered 56% of the variance in VO_2_peak (*R* ^2^ = 0.56, F (1, 61) = 79.2, *p* < 0.001), and OUES_75_ significantly predicted VO_2_peak (*β* = 0.75, *p* < 0.001). The RMSE was 0.32 L·min^−1^.

(c) VO_2_peak = 0.96 + 0.66 · OUES_100_

The regression model covered 62% of the variance in VO_2_peak (*R* ^2^ = 0.62, F (1, 61) = 100.1, *p* < 0.001), and OUES_75_ significantly predicted VO_2_peak (*β* = 0.79, *p* < 0.001). The RMSE was 0.30 L·min^−1^.

**FIGURE 3:**
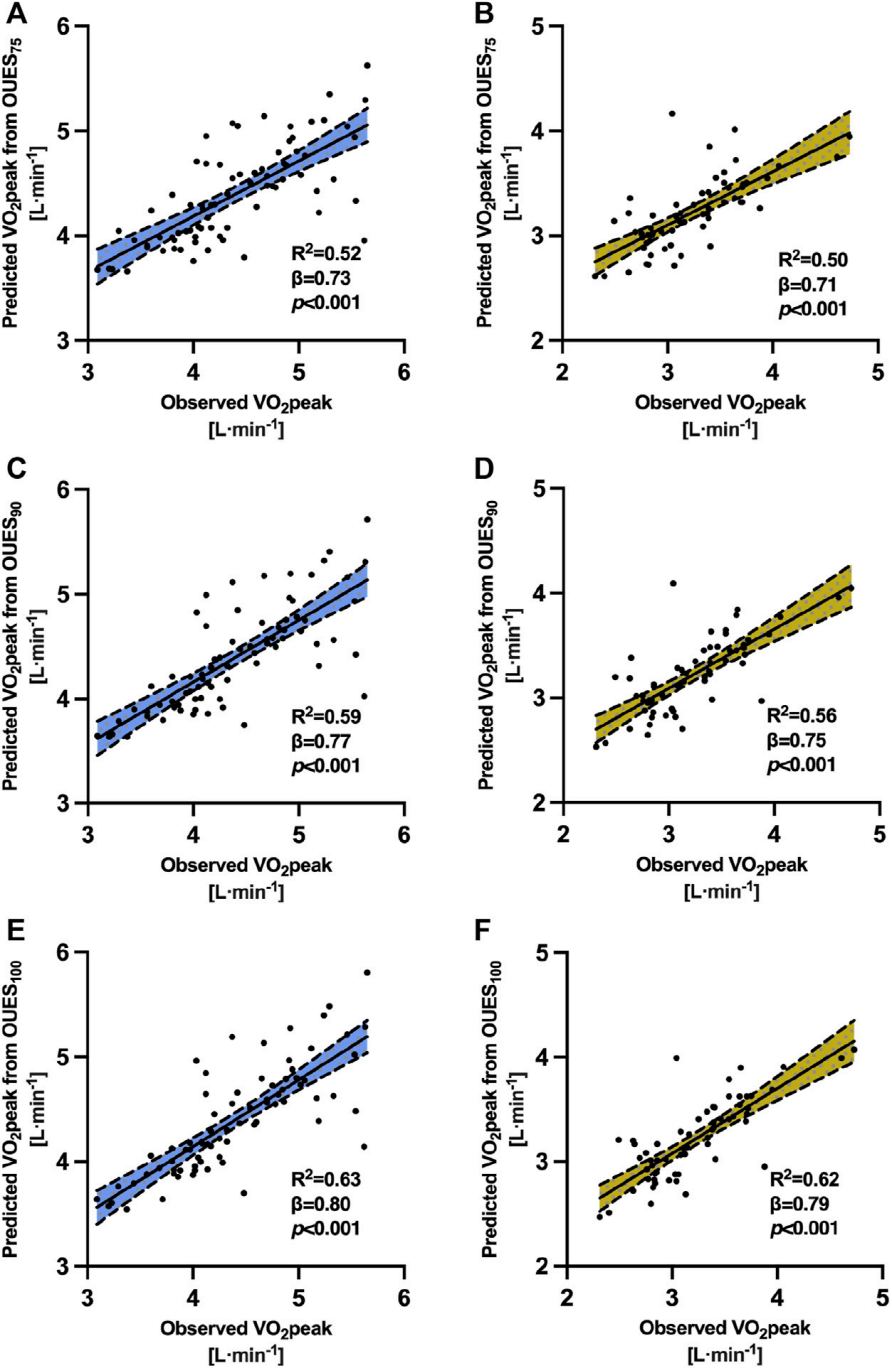
Prediction power of oxygen uptake efficiency slope on peak oxygen uptake. Abbreviations: VO_2_peak, peak oxygen uptake; *R* ^2^, adjusted coefficient of determination; OUES_75_, oxygen uptake efficiency slope from 75% of exercise duration; OUES_90_, oxygen uptake efficiency slope from 90% of exercise duration; OUES_100_, oxygen uptake efficiency slope from 100% of exercise duration. Note. **(A)** Prediction power of the oxygen uptake efficiency slope from 75% of exercise duration on VO_2_peak for male subjects. **(B)** Prediction power of the oxygen uptake efficiency slope from 75% of exercise duration on VO_2_peak for female subjects. **(C)** Prediction power of the oxygen uptake efficiency slope from 90% of exercise duration on VO_2_peak for male subjects. **(D)** Prediction power of the oxygen uptake efficiency slope from 90% of exercise duration on VO_2_peak for female subjects. **(E)** Prediction power of the oxygen uptake efficiency slope from 100% of exercise duration on VO_2_peak for male subjects. **(F)** Prediction power of the oxygen uptake efficiency slope from 100% of exercise duration on VO_2_peak for male subjects. In each model, the regression was significant with *p* < 0.001. The *x*-axis presents directly measured VO_2_peak in cardiopulmonary exercise tests. The *y*-axis presents predicted VO_2_peak by oxygen uptake efficiency slopes from 75%, 90%, and 100% portions of exercise data. The continuous central line represents a trend line. The upper and lower dashed lines represent upper and lower 95% limits of normal.

Plots visualizing the prediction powers are shown in Figure 3.

### Developed prediction equations for OUES

We predicted OUES using several variables: age, sex, and BSA. Models for OUES_75_, OUES_90_, and OUES_100_ showed similar and promising accuracy. In the developed equations, BSA should be in m^2^, age in years, and the sex coefficients are 2 if female and 1 if male. The RMSE was similar in all three equations (0.72–0.77 mL·min^−1^/L·min^−1^) developed to predict OUES obtained from different portions of the exercise data.

The new prediction equations for OUES are as follows:

(a) OUES_75_ = 1.09 + 2.87 · BSA—0.0030 · (age · sex)

The prediction equation explained 34% of variance in OUES_75_ (R = 0.59, *R* ^2^ = 0.34, F (2, 137) = 36.1, *p* < 0.001). The overall RMSE was 0.77 mL·min^−1^/L·min^−1^.

(b) OUES_90_ = 1.46–3.02 · BSA—0.0010 · (age · sex)

The prediction equation explained 35% of variance in OUES_75_ (R = 0.60; *R* ^2^ = 0.35, F (2, 137) = 37.5, *p* < 0.001). The overall RMSE was 0.77 mL·min^−1^/L·min^−1^.

(c) OUES_100_ = 1.54–2.99 · BSA—0.0014 · (age · sex)

The prediction equation explained 36% of variance in OUES_75_ (R = 0.61; *R* ^2^ = 0.36, F (2, 137) = 40.3, *p* < 0.001). The overall RMSE was 0.72 mL·min^−1^/L·min^−1^.

### Model validation

**FIGURE 4:**
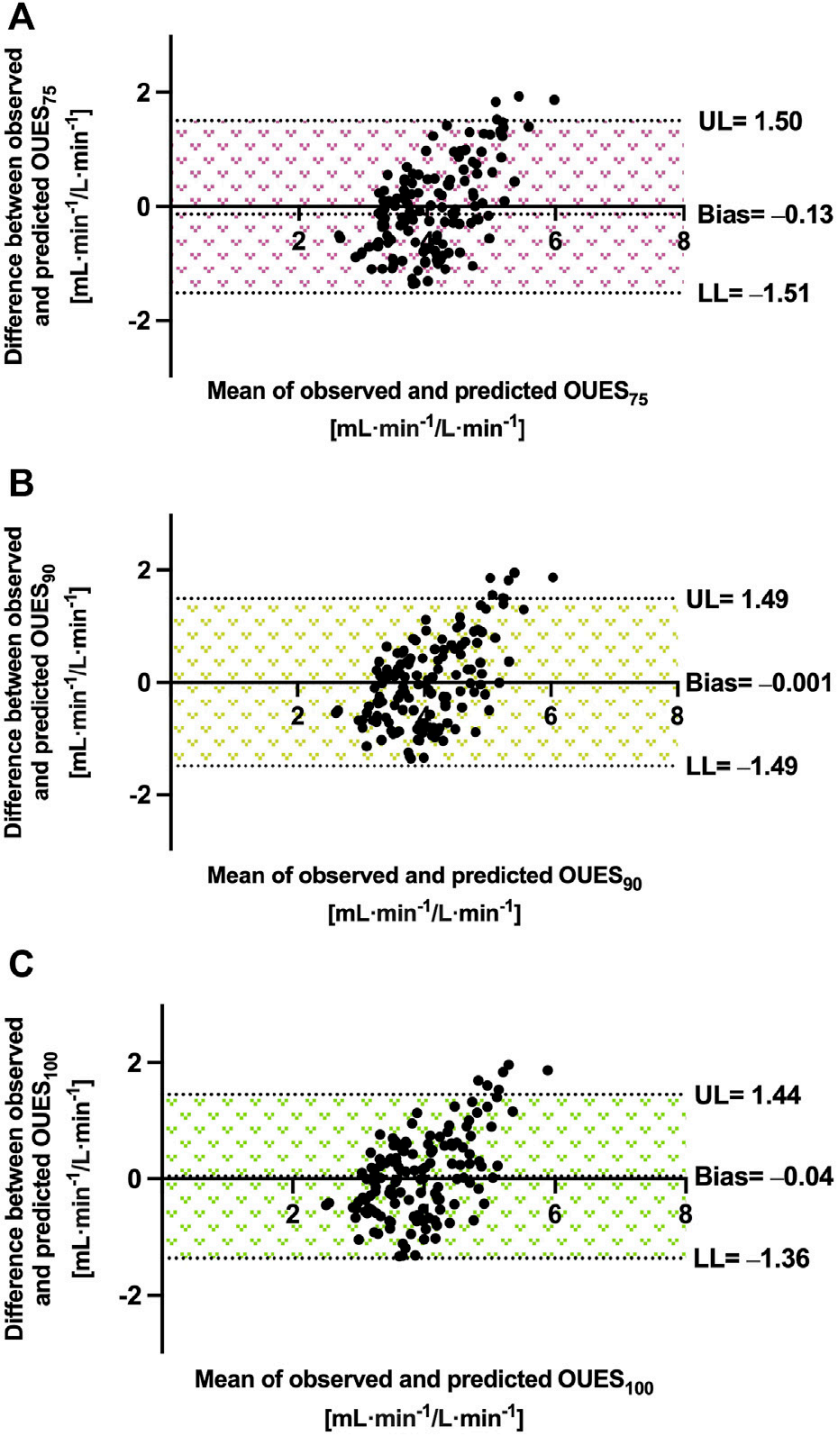
Precision of OUES equations in endurance athletes. Abbreviations: OUES_75_, oxygen uptake efficiency slope from 75% of exercise duration; OUES_90_, oxygen uptake efficiency slope from 90% of exercise duration; OUES_100_, oxygen uptake efficiency slope from 100% of exercise duration; UL, upper 95% limit of agreement; LL, lower 95% limit of agreement. Note: the means and differences are calculated in mL·min^−1^/L·min^−1^. **(A)** Prediction performance for OUES_75_. **(B)** Prediction performance for OUES_90_. **(C)** Prediction performance for OUES_100_. The area between LL and UL represents the limit of agreement and is indicated as the measure of the model’s fit.

In all external models, a high variability was observed between the observed and predicted data sets (only 4 out of 11 models have ICC>0.5). The best alignment was noted in Milani et al. (2023) (equation adjusted to age) (ICC = 0.510 [0.491, 0.529] for OUES_75_, ICC = 0.512 [0.492, 0.531] for OUES_90_, and ICC = 0.529 [0.511, 0.548] for OUES_100_). The equations underestimated all OUES_75_, OUES_90_, and OUES_100_. Underestimation ranged up to 186.8% for the formula proposed by Marinov et al. (2007). However, the overall prediction trend in endurance athletes was maintained. The predicted values followed those directly observed, but the degree of explained variance was wide (*R* ^2^ = 0.004–0.388). For detailed external validation, see Table 3. The agreement was lower in equations for OUES_90_ (*R* ^2^ = 0.290) and OUES_75_ (*R* ^2^ = 0.346). Bland–Altman plots showing the validity of our models are shown in Figure 4. All the equations slightly underestimated OUES. The best-performing model was the one for OUES_90_, with the bias of only −0.001 mL·min^−1^/L·min^−1^. The next was the model for OUES_100_ (bias = −0.04 mL·min^−1^/L·min^−1^). The least-performing model was for OUES_75_, with an error of −0.13 mL·min^−1^/L·min^−1^. The limits of agreement were narrow and similar in all models. For OUES_75_, it ranged between −1.51 and 1.50 mL·min^−1^/L·min^−1^. The limit of agreement in the equation for OUES_90_ was from −1.49 to 1.49 mL·min^−1^/L·min^−1^. Finally, in the model for OUES_100_, it ranged between −1.36 and 1.44 mL·min^−1^/L·min^−1^. As presented in Figure 4, some athletes exceeded the upper limits of agreement, but no one exceeded the lower limits.

**TABLE 3:** Reliability of OUES prediction models.

Reference model		OUES_75_					OUES_90_					OUES_100_				
		ICC	95% CI		*R* ^2^	% Predicted	ICC	95% CI		*R* ^2^	% Predicted	ICC	95% CI		*R* ^2^	% Predicted
			LL	UL				LL	UL				LL	UL		
Hollenberg and Tager (2000)		0.505	0.486	0.524	0.289	142.4	0.506	0.486	0.525	0.289	142.1	0.524	0.505	0.542	0.300	138.8
Buys et al. (2015)	1	0.431	0.411	0.453	0.267	127.5	0.431	0.410	0.452	0.264	127.2	0.449	0.428	0.469	0.272	124.2
	2	0.488	0.469	0.508	0.310	131.0	0.490	0.470	0.509	0.311	130.7	0.510	0.491	0.529	0.323	127.7
	3	0.493	0.474	0.513	0.311	131.1	0.495	0.475	0.514	0.312	130.8	0.515	0.496	0.534	0.324	127.7
Milani et al. (2023)	1	0.510	0.491	0.529	0.298	153.7	0.512	0.492	0.531	0.299	153.4	0.529	0.511	0.548	0.310	149.8
	2	0.062	0.037	0.088	0.004	160.0	0.064	0.038	0.090	0.004	159.6	0.066	0.040	0.092	0.004	155.9
	3	0.229	0.205	0.254	0.064	153.2	0.234	0.210	0.259	0.067	152.8	0.246	0.221	0.270	0.069	149.2
Ashikaga et al. (2021)		0.332	0.309	0.355	0.261	179.8	0.331	0.308	0.354	0.258	179.3	0.348	0.325	0.371	0.266	175.1
Sun et al. (2012)		0.447	0.426	0.468	0.241	137.3	0.446	0.425	0.467	0.240	137.1	0.464	0.444	0.484	0.249	133.8
Marinov et al. (2007)		0.321	0.298	0.344	0.188	186.8	0.326	0.303	0.349	0.193	186.3	0.350	0.327	0.373	0.209	181.9
Marinov and Kostianev. (2003)		0.443	0.422	0.463	0.357	134.6	0.451	0.430	0.472	0.369	134.2	0.477	0.457	0.497	0.388	131.0

Abbreviations: OUES_75_, oxygen uptake efficiency slope from 75% of exercise duration; OUES_90_, oxygen uptake efficiency slope from 90% of exercise duration; OUES_100_, oxygen uptake efficiency slope from 100% of exercise duration; ICC, interclass correlation coefficient; CI, confidence interval; LL, lower limit; UL, upper limit; *R* ^2^, coefficient of determination.Note: coefficient of determination (*R* ^2^) is considered as the adjusted *R* ^2^. OUES_75_ was 3.50 ± 0.65 mL·min^−1^/L·min^−1^ for female subjects, 4.53 ± 0.90 mL·min^−1^/L·min^−1^ for male subjects, and 4.07 ± 0.95 mL·min^−1^/L·min^−1^ for the total population. OUES_90_ was 3.49 ± 0.62 mL·min^−1^/L·min^−1^ for female subjects, 4.52 ± 0.91 mL·min^−1^/L·min^−1^ for male subjects, and 4.06 ± 0.95 mL·min^−1^/L·min^−1^ for the total population. OUES_100_ was 3.41 ± 0.58 mL·min^−1^/L·min^−1^ for female subjects, 4.41 ± 0.87 mL·min^−1^/L·min^−1^ for male subjects, and 3.96 ± 0.90 mL·min^−1^/L·min^−1^ for the total population. All reference models underestimated OUES. Significant *p*-values (<0.05) were marked with *, while *p*-values (<0.001) were marked with †.

## Discussion

We have shown that OUES is a valuable submaximal parameter in elite athletes as well. The key findings of this study are as follows:

• First, the new prediction equations provide promising but moderate precision for endurance athletes and explain between 34% and 36% of the variance in OUES.
• Second, the overall association between OUES_75_, OUES_90_, and OUES_100_ was similar to those observed in untrained and elderly subjects.
• Third, we observed a strong relationship between OUES and VO_2_peak, even in well-trained endurance athletes, and its predictive impact was comparable for all time intervals.
• Finally, the findings from this study confirm our hypothesis that prediction equations derived from the general population perform poorly in a cohort of endurance athletes.

We noticed that even when OUES is elevated in high-performance endurance athletes, the overall trend and lack of significant differences were still maintained. Previous studies have not assessed such relationships in trained participants or merged trained and untrained subjects (Akkerman et al., 2010; Sun et al., 2012). We confirmed that even if an athlete can continue strenuous exercises for a prolonged time, the OUES remains stable across its duration. On the other hand, when one of the time intervals (OUES_75_, OUES_90_, or OUES_100_) is underestimated or overestimated and presents a high difference from others, it should raise awareness (Hollenberg and Tager, 2000). Thus, monitoring OUES can valuably contribute to the assessment of the response profile to exercise (Hammond and Froelicher, 1985; Akkerman et al., 2010). Medical professionals can add OUES to their testing portfolio when challenging CPET results in this unique patient population.

Furthermore, our results show that OUES is a comparable predictor of VO_2_peak independently from the measuring interval. In univariable models, we noticed that the relationship remained very strong, regardless of sex, age, and other covariates. For all prediction models, the overall regression was statistically significant (*R* ^2^ = 0.50–0.63, all *p* < 0.001), and OUES showed high prediction abilities (*β* = 0.71–0.80, all *p* < 0.001). Both submaximal OUES_75_ and OUES_90_ explained up to 60% of the variance in VO_2_peak. Previously, Brown et al. reported a weaker correlation between OUES and VO_2_peak, with *R* ^2^ = 0.09–0.15 (Brown et al., 2013). Perhaps, their research was conducted on youngsters (mean age was 14.7 years) and with fewer athletes (n = 25) than in ours (mean age 22.3 years, n = 140). The present study strongly advocates the conclusions made by Sun et al. that OUES and VO_2_peak measure analogous mechanisms in athletes (Sun et al., 2012). Each may be complementary and supplementary to the other.

External validation of the prediction models for OUES confirmed the results of our previous studies that equations derived from the general population showed limited agreement when applied to endurance athletes (Wiecha et al., 2023a; Kasiak et al., 2023). Although the general trend was maintained, the variability between the observed and predicted values was high. Only 4 of the 11 models achieved at least ICC ≥0.5, which is considered the lowest borderline of moderate compliance (for precise data, see Table 3) (Koo and Li, 2016). Adjusting models to BSA or weight provides some additional benefits. The simple equations by Marinov and Kostianev (2003) using only BSA covered the highest amount of variance (*R* ^2^ = 0.388). The model by Marinov and Kostianev (2003) is a model derived from a pediatric sample. The athletic cohort of this study consisted of younger individuals with a mean age of roughly 22.3 years. We stipulate that this is the underlying reason for why the use of the age covariate provided only a slight predictive value in our participants. However, future studies should confirm our findings and externally examine our equations as this is the first study prediction of OUES among endurance athletes.

We adjusted OUES to BSA, as recommended by Baba et al. (1996) and Hollenberg and Tager (2000). Our model performed more accurately or with similar precision than that proposed by previous authors. The *R* ^2^ was approximately 34%–36%. Comparably, Milani et al. (2023) observed *R* ^2^ between 7.7% and 10.4%. A study by Milani et al. (2023) was conducted among healthy individuals, although with wider age ranges and with other ethnicity than in our participants. A model partially derived from trained subjects by Sun et al. (2012) provided R = 0.701, which was slightly higher than the R-value achieved in this study (between 0.59 and 0.61). To enrich the quality of the analysis, we additionally validated each of our models. It is worth underlining that our models performed comparably during the development and validation. The mean bias was −0.13 mL·min^−1^/L·min^−1^ for the predicted OUES_75_, –0.001 mL·min^−1^/L·min^−1^ for OUES_90_, and –0.04 mL·min^−1^/L·min^−1^ for OUES_100_. This shows that the model fits well, as presented in Figure 2.

### Perspectives and significance

So far, the diagnostic value of submaximal cardiorespiratory fitness has been mostly discussed in clinical populations when the maximal strenuous effort is not recommended, e.g., in heart failure (Metra et al., 1998). However, among healthy, highly trained individuals, the holistic approach should include an evaluation of the whole exercise duration (Shushan et al., 2022). Submaximal performance could also deteriorate in elite endurance athletes, e.g., after a viral infection (Sliz et al., 2022). Sheridan et al. noticed limited prediction power of OUES for VO_2_peak when measured from the start to the ventilatory threshold in endurance athletes (Sheridan et al., 2021). OUES measured from the percentage of exercise duration is a more objective and replicable index because it includes the precisely truncated parts of exercise data (in our study, 75%, 90%, and 100%, respectively) (Baba, 2000). Even in elite athletes, OUES links well with the most reliable index of cardiorespiratory fitness, i.e., VO_2_peak. Therefore, considering OUES as a prognostic indicator is justified when we suspect CVD in the athletic population. Furthermore, comparing OUES of a single participant between 75%, 90%, and 100% intervals would be beneficial to determine how an athlete’s heart responds to increasing intensity. Furthermore, the developed new prediction equations could be applied when direct CPET is not possible.

### Limitations and interpretation

Our study also has some points that have to be mentioned to ensure correct interpretation. The population was homogenous in age and ethnicity. The other studies for OUES prediction models included a higher number of participants than our study. In this research, the average age of the participants was roughly 22 years. The subjects were younger and had above-average endurance capacity when compared to other studies in this field. Our athletes were healthy. Thus, the results have limited application in patients with pathologies. We conducted CPET on cycle ergometry. Testing modality could influence the achieved performance, and exercise tests performed on other machines (treadmill and rowing ergometry) could provide slightly different results (Price et al., 2022). The participants usually achieve a lower performance on the cycle ergometer than on the treadmill, and our models are mostly tailored for cycling exercise tests. We expect that applying the presented models to other testing protocols could cause some inaccuracies. Therefore, we recommend considering all the above-mentioned aspects when deriving future reference equations. This is the first study of OUES predictions in highly fit participants, so we recommend careful extrapolation when considering the results. The external validation of our equations is welcome. Moreover, the development of formulas from other testing machines is also needed.

## Conclusion

OUES remains stable between 75%, 90%, and 100% of exercise duration even in highly trained individuals. OUES was comparable between the measurements for endurance athletes. The prediction models derived from the general population demonstrated poor alignment. We do not recommend them for endurance athletes. New equations explained up to 36% of the variance and were inaccurate for only up to −0.13 mL·min^−1^/L·min^−1^. The proposed equations are internally validated and designed for endurance athletes. Medical professionals and physicians should be acknowledged to precisely adjust the training and properly interpret the specific cardiovascular response profile in athletes.

## Data Availability

The raw data supporting the conclusion of this article will be made available by the authors, without undue reservation.
